# Placental macrophage responses to viral and bacterial ligands and the influence of fetal sex

**DOI:** 10.1016/j.isci.2022.105653

**Published:** 2022-11-22

**Authors:** Paschalia Pantazi, Myrsini Kaforou, Zhonghua Tang, Vikki M. Abrahams, Andrew McArdle, Seth Guller, Beth Holder

**Affiliations:** 1 Institute of Reproductive and Developmental Biology, Department of Metabolism, Digestion, and Reproduction, Imperial College London, London W12 0HS, UK; 2 Section of Paediatric Infectious Disease, Department of Infectious Disease, Imperial College London London W2 1NY, UK; 3 Department of Obstetrics, Gynecology and Reproductive Sciences, Yale School of Medicine, New Haven, CT 06510, USA

**Keywords:** Immunology, immune response, microbiology, proteomics

## Abstract

Bacterial and viral infections of the placenta are associated with inflammation and adverse pregnancy outcomes. Hofbauer cells (HBCs) are fetal-origin macrophages in the placenta, proposed to protect the fetus from vertical pathogen transmission. We performed quantitative proteomics on term HBCs under resting conditions and following exposure to bacterial and viral pathogen-associated molecular patterns (PAMPs), and investigated the contribution of fetal sex. Resting HBCs expressed proteins pertinent to macrophage function, including chemokines, cytokines, Toll-like receptors, and major histocompatibility complex class I and II molecules. HBCs mounted divergent responses to bacterial versus viral PAMPs but exhibited protein expression changes suggestive of a more pro-inflammatory phenotype. A comparison between male and female HBCs showed that the latter mounted a stronger and wider response. Here, we provide a comprehensive understanding of the sex-dependent responses of placental macrophages to infectious triggers, which were primarily associated with lipid metabolism in males and cytoskeleton organization in females.

## Highlights

• HBCs exhibit a pro-inflammatory phenotype in response to LPS and PIC
• HBCs employ distinct defense mechanisms against LPS and PIC
• Female HBC responses are associated with cytoskeleton organization
• Male HBC responses are associated with lipid metabolism

## Graphical abstract

**Figure:**
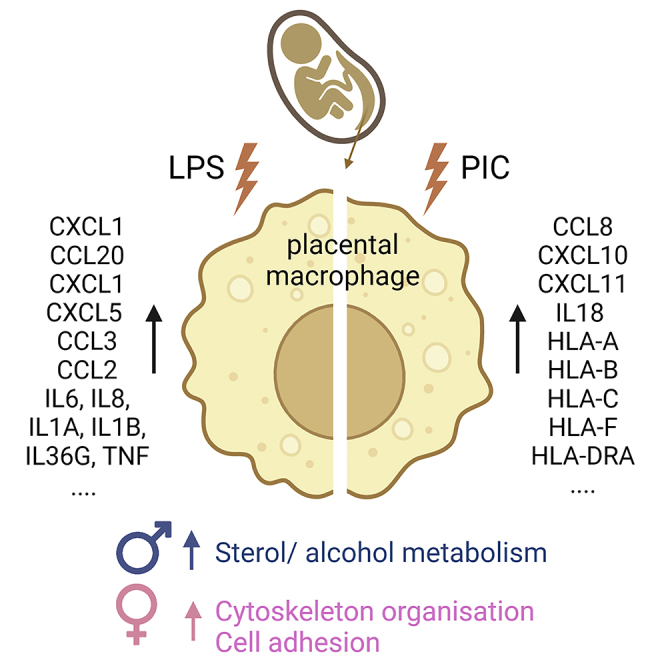

## Introduction

Placental macrophages, termed Hofbauer cells (HBCs), are the only immune cell subset found in human placental villous tissue.^1^ These fetal-origin macrophages are present throughout gestation and contribute to the immune protection of the fetus during pregnancy.^2^ HBCs also help maintain homeostatic conditions in the placenta by regulating processes such as angiogenesis,^3^ vasculogenesis,^4^ and tissue remodeling,^5^ consistent with their characterization as anti-inflammatory M2-like macrophages.^3^^,^^6^

HBCs are highly plastic, allowing them to adapt their phenotype in response to environmental signals. In pregnancy complications, such as pre-eclampsia and chorioamnionitis, HBCs change in their number and phenotype.^7^^,^^8^^,^^9^^,^^10^ HBCs respond to, and are targets for, several pathogens (reviewed in Fakonti et al., 2020).^11^ HBCs release pro-inflammatory cytokines, activate NF-κB signaling, and upregulate M1 pro-inflammatory immunophenotype-associated genes upon encounter with whole bacteria or bacterial pathogen-associated molecular patterns (PAMPs) *in vitro*.^12^^,^^13^^,^^14^ Moreover, HBCs fight bacterial infection by releasing extracellular traps - protrusions containing metalloproteases -^15^ as well as undergoing pyroptosis, a form of inflammatory programmed cell death involving inflammasome-associated caspase-1.^14^ First-trimester HBCs have the capacity to proliferate *in situ* in response to bacterial ligands, phagocytosing *Escherichia coli* ligands and microspheres.^16^ HBCs also respond to a range of viruses. For example, Hendrix et al., demonstrated that HBCs infected with γ-herpesvirus, MHV-68, *in vitro* release the pro-inflammatory cytokine interleukin (IL)-1β, activating endothelial cells toward a pro-neutrophilic response.^17^

In contrast to these studies of HBC infection control, several other studies suggest that HBCs either do not respond or respond in favor of pathogen dissemination. For example, Schliefsteiner et al., supported that HBCs resist bacterial cues and do not change their M2-like phenotype, despite producing tumor necrosis factor-α (TNF-α) and interferon γ (IFN-γ) in response to bacterial PAMPs.^18^ HBCs have also been reported to act as reservoirs for replication and transmission of the respiratory syncytial virus *in vitro*.^19^ Furthermore, HBC co-infection with cytomegalovirus has been shown to enhance the replication of HIV-1.^20^

A recent study profiled the gene expression of placental macrophages from first-trimester placentas along with maternal blood and immune cells from the decidua, using spatiotemporal single-cell RNA sequencing (scRNAseq), developing a single-cell atlas of the maternal-fetal interface containing information on cell location and cell-cell interactions in the decidua and placenta.^21^ But despite numerous studies looking at HBCs in placental samples from infected mothers, or the *in vitro* response of HBCs to pathogens, a comprehensive understanding of the functional role of HBCs during infection is still lacking. Most studies have looked at a small number of specific surface proteins, or cytokine release, rather than an untargeted approach. Finally, although the influence of sex in immunological responses is increasingly acknowledged,^22^ and sex differences in placental genes, proteins, and function have been reported,^23^^,^^24^^,^^25^ there is a dearth of knowledge regarding the potential association between fetal sex and HBC phenotype and function.

In this study, we sought to comprehensively characterize term HBC phenotype and responses to pathogens and the contribution of fetal sex to these responses. Utilizing quantitative proteomics, we found that term HBCs contain a number of immune-related proteins including cytokines, chemokines, Toll-like receptors (TLRs), and scavenger receptors. We show that HBCs mount divergent responses to bacterial versus viral pathogen-associated patterns (PAMPs), but exhibit phenotypic changes suggestive of an M2-like to M1-like switch in response to both. Finally, we provide the first clear picture of sex-dependent differences in HBC phenotypes and responses, primarily related to lipid metabolism in males, and cytoskeleton organization in females. These findings provide a novel understanding of the phenotype of term placental macrophages, and their sex-dependent responses to infectious triggers.

## Results

### Patient information

A total of ten placentas were included in this study; with a 50:50 balance of male and female fetal sex (Table S1). All subjects were White and non-Hispanic, and all deliveries were elective cesarean sections, without labor, with a median gestational age at delivery of 39 weeks + 1day.

### Proteomic profile of term placental macrophages

**Figure 1:**
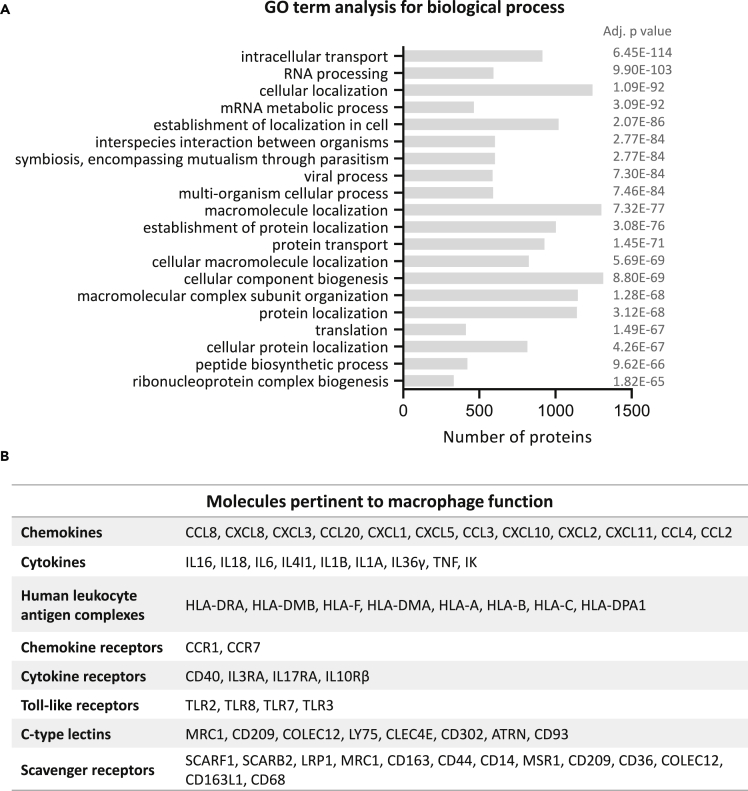
Characterization of placental macrophages The proteome of non-treated term HBCs was investigated. (A) The proteins were analyzed by DAVID functional annotation to produce clusters (≥2 proteins/cluster) and Gene ontology (GO) terms corresponding to biological process (GOTERM_BP_FAT) were extracted. The histogram shows the top 20 GO terms significantly associated (adj. p ≤ 0.05) with the protein list along with the number of proteins in each cluster. (B) List(s) of macrophage-relevant proteins present in resting HBCs.

A total of 5892 protein IDs were identified in our quantitative proteomic analysis of HBCs (Data S1). Functional enrichment analysis of these proteins revealed their involvement in metabolic processes, viral processes, antigen processing and presentation, membrane disassembly, cell cycle phase transition, cytokine production, and gene expression amongst other biological processes (Figure 1A and Data S1). Interrogation of their molecular function found that these proteins were primarily RNA-, protein-, cell adhesion molecule-, and enzyme-binding. Cellular component analysis revealed the association of these proteins with membrane-bound vesicles, extracellular region parts, cell junctions, and cytosol (Data S1). HBCs expressed a range of proteins pertinent to macrophage function (Figure 1B), such as chemokines (CCL8, CXCL8, CXCL3, CCL20, CXCL1, CXCL5, CCL3, CXCL10, CXCL2, CXCL11, CCL4, CCL2) and chemokine receptors (CCR1, CCR7), cytokines (IL16, IL18, IL6, IL4I1, IL1B, IL1A, IL36G, TNF-α, IK) and cytokine receptors (CD40, IL3RA, IL17RA, IL10Rβ), as well as Toll-like receptors 2, 3, 7 and 8 (TLR2, TLR3, TLR7, TLR8). Term HBCs were positive for classical major histocompatibility complex (MHC) class I (HLA-A, HLA-B, HLA-C), non-classical MHC class I (HLA-F), and MHC class II (HLA-DRA, HLA-DMB, HLA-DMA, HLA-DPA1) molecules. Moreover, they have several scavenger receptors; surface molecules that typically bind multiple ligands to remove nonself or altered-self targets.^26^ Finally, we found previously identified Hofbauer cell markers such as CD163, CD68, CD209, FOLR2, ARG2, VSIG4, and MRC1.^6^^,^^16^

### Changes in the proteomic profile of placental macrophages in response to bacterial lipopolysaccharide

**Figure 2:**
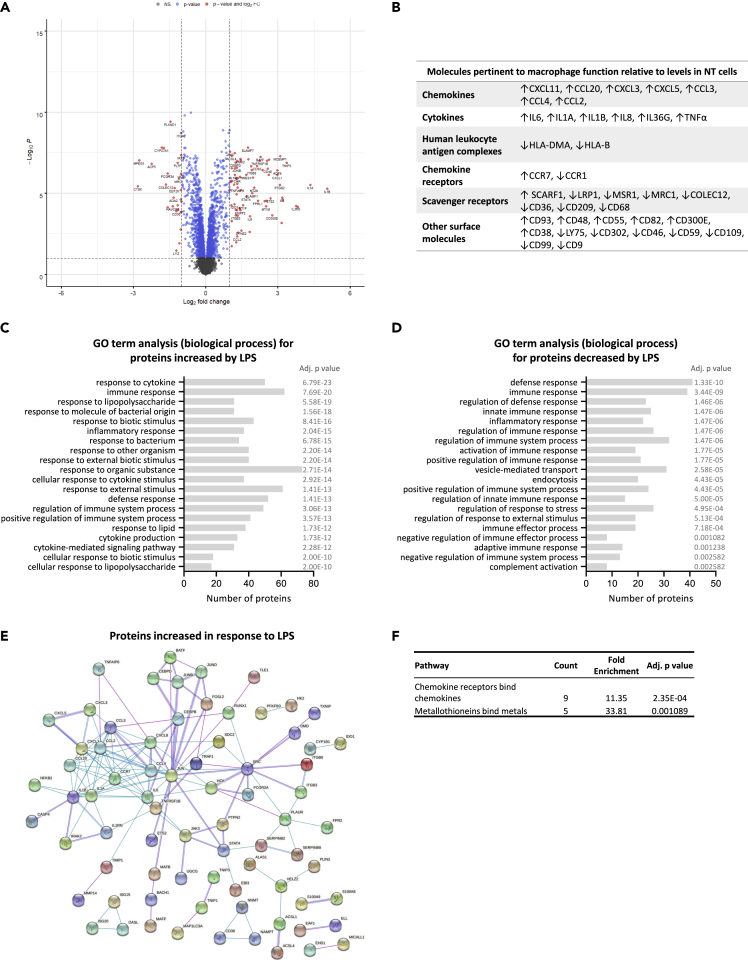
Proteomic characterization of placental macrophages in response to bacterial PAMP HBCs were exposed to bacterial lipopolysaccharide (LPS) for 24h, followed by TMT-proteomics, to enable the quantitative measurement of the LPS response. (A) Volcano plot depicting the log2 fold differences in protein abundance between LPS-treated and non-treated HBCs. Increased (FC > 1.5) and decreased (FC < −1.5) proteins are labeled green and are found on the right and the left side of the graph respectively. A cut-off of 0.05 was applied for the adjusted p values (adj. p value; y axis). (B) Proteins pertinent to macrophage function found up (↑) or down (↓) in LPS-treated HBCs compared with non-treated (NT) cells. (C and D) Gene ontology (GO) analysis of the (C) increased (FC > 1.5, adj. p value < 0.05) and (D) decreased (FC < −1.5, adj. p value < 0.05) proteins in HBC treated with LPS compared with controls (non-treated HBCs). The proteins were analyzed by DAVID functional annotation to produce clusters (≥2 proteins/cluster) and GO terms corresponding to biological process (GOTERM_BP_FAT) were extracted. The histogram shows the top 20 GO terms significantly associated (adj p ≤ 0.05) with the protein list along with the number of proteins in each cluster. (E) Interaction networks of the up-regulated proteins using STRING v11.5. Colored lines between the proteins indicate the interaction evidence (pink: experimentally determined, blue: from curated databases), minimum required interaction score - medium confidence (0.4), disconnected nodes are hidden from the graph. (F) Reactome pathway enrichment analysis. Two pathways were over-represented (adj. p value < 0.05) in LPS-treated HBCs. For each pathway, the number of proteins (Count) and the (Fold Enrichment) are given.

To determine their response to bacterial infection, we treated HBCs with bacterial LPS. A total of 314 proteins were changed by at least 1.5-fold (adj. p < 0.05) in the LPS-treated HBCs compared with NT, with 176 being increased and 138 decreased (Figure 2A) (Data S2). Among the decreased proteins were MRC1 and CD209, markers of M2 anti-inflammatory macrophages, while CCR7, a marker of M1 pro-inflammatory macrophages, was upregulated (Figure 2B),^27^ suggesting that HBCs may move away from their M2-like phenotype upon encounter with bacteria. Gene ontology analysis of the LPS-induced differentially abundant proteins revealed their involvement in the immune response, inflammatory response, and response to external stimulus (Figures 2C and 2D). More specifically, Reactome analysis of the LPS-increased proteins identified two pathways: “chemokine receptors bind chemokines” (proteins involved: CCL20, CXCL8, CXCL1, CCL3, CXCL5, CCL2, CCL4, CCR7, CXCL3) and “metallothioneins bind metals” (proteins involved: MT1M, MT1X, MT2A, MT1E, MT1F) (Figures 2E and 2F). No pathways were identified by Reactome analysis of the LPS-decreased proteins (adj. p value < 0.05). Inter-Pro analysis, which classifies proteins into families according to their domains and important sites, showed that several LPS-upregulated proteins bear basic leucine zipper domains, chemokine conserved sites, metallothionein domains, and DNA-binding sites (Data S2). Regarding the proteins decreased by LPS treatment, the top significantly changed families were immunoglobulin E, immunoglobulin-like, and MD-2-related lipid-recognition domains (Data S2).

### Changes in proteomic profile of placental macrophages in response to viral dsRNA

**Figure 3:**
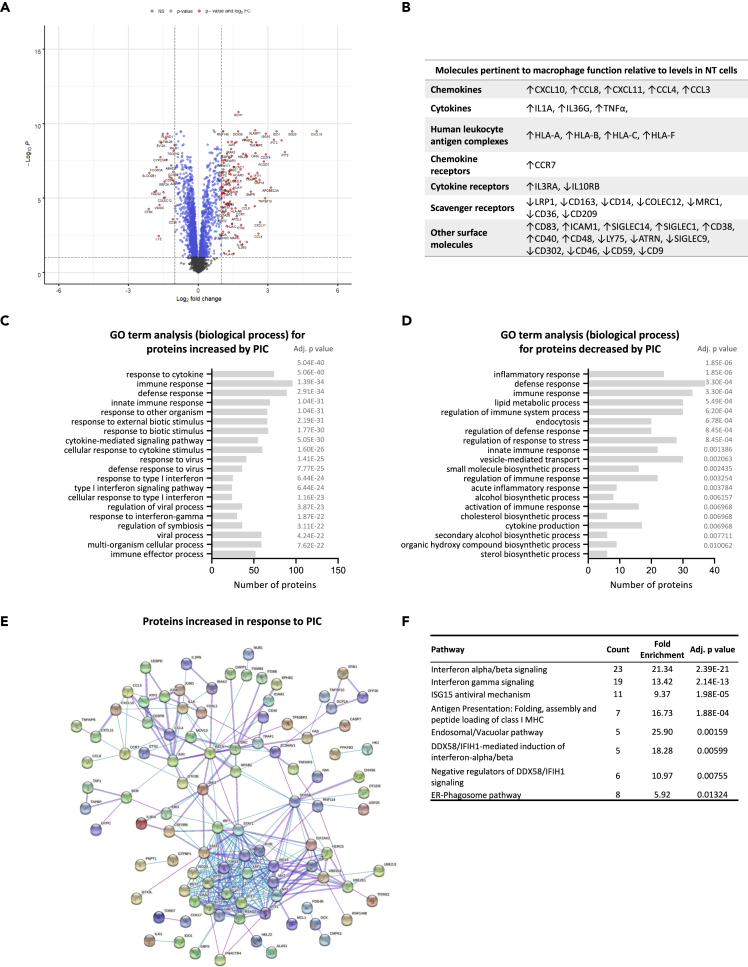
Proteomic characterization of placental macrophages in response to viral PAMP HBCs were exposed to poly(I:C) (PIC), a viral RNA analog, for 24h, followed by TMT-proteomics, to enable the quantitative measurement of the PIC response. (A) Volcano plot depicting the fold differences in protein abundance between PIC-treated and non-treated HBCs. Increased (FC > 1.5) and decreased (FC < −1.5) proteins are labeled green and are found on the right and the left side of the graph respectively. A cut-off of 0.05 was applied for the adjusted p values (adj. p value, y axis). (B) Proteins pertinent to macrophage function found up (↑) or down (↓) regulated in PIC-treated HBCs. (C and D) Gene ontology (GO) analysis of the (C) up-regulated (FC > 1.5, adj. p value < 0.05) and (D) down-regulated (FC < −1.5, adj. p value < 0.05) proteins in HBC treated with PIC compared with controls (non-treated HBCs). The proteins were analyzed by DAVID functional annotation to produce clusters (≥2 proteins/cluster) and GO terms corresponding to biological process (GOTERM_BP_FAT) were extracted. The histogram shows the top 20 GO terms significantly associated (adj p ≤ 0.05) with the protein list along with the number of proteins in each cluster. (E) Interaction networks of the up-regulated proteins using STRING v11.5. Colored lines between the proteins indicate the interaction evidence (pink: experimentally determined, blue: from curated databases), minimum required interaction score - medium confidence (0.4), disconnected nodes are hidden from the graph. (F) Reactome pathway enrichment analysis. A total of 8 pathways were over-represented (adj. p value < 0.05) in PIC-treated HBCs. For each pathway, the number of proteins (count) and the fold enrichment are given.

To investigate HBC responses to viral infection, we treated cells with poly(I:C) (PIC) a viral dsRNA mimic. A total of 393 proteins were changed by at least 1.5-fold (p < 0.05) in the PIC-treated HBCs compared with NT, with 240 being increased and 153 decreased (Figure 3A) (Data S3). Similarly to LPS, PIC caused a reduction in the expression of the M2 markers MRC1, CD163, and CD209, and an increase of the M1 markers CXCL11 and IL1A^27^ (Figure 3B), suggesting that HBCs may become more pro-inflammatory upon virus encounter. Gene ontology analysis of the proteins increased in response to PIC indicated their involvement in biological processes including antigen processing and presentation, viral processes, cell activation, cell death, regulation of cytokine-mediated signaling, and protein transport (Figure 3C). On the other hand, the proteins that were decreased in the PIC-treated HBCs were involved in cytokine production, immune effector processes, extracellular matrix organization, metabolic processes, response to external stimulus, and endocytosis (Figure 3D). Pathway enrichment analysis revealed the induction of several Reactome pathways in HBCs in response to PIC treatment: “interferon alpha/beta signaling,” “interferon gamma signaling,” “ISG15 antiviral mechanism,” “antigen presentation: folding, assembly and peptide loading of class I MHC,” “endosomal/vacuolar pathway,” “DDX58/IFIH1-mediated induction of interferon-alpha/beta,” “negative regulators of DDX58/IFIH1 signaling,” and “ER-phagosome pathway” (Figures 3E and 3F).

### Differential response to bacterial and viral pathogen-associated molecular patterns by placental macrophages

**Figure 4:**
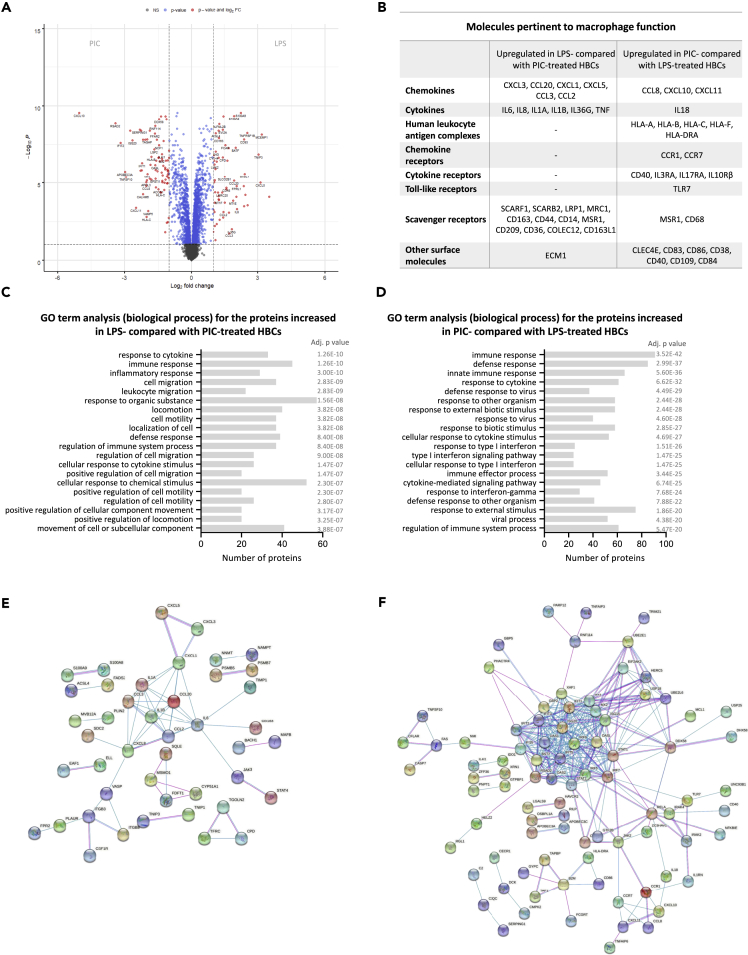
Differential placental macrophage responses to bacterial and viral PAMPs (A) Volcano plot depicting the fold differences in protein abundance between LPS-treated and PIC-treated HBCs. Proteins increased (FC > 1.5) in LPS-treated HBCs are found on the right side of the graph, while proteins decreased (FC < −1.5) in LPS-treated and therefore increased in PIC-treated HBCs are found on the left side. A cut-off of 0.05 was applied for the adjusted p values (adj. p value, y axis). (B) Proteins pertinent to macrophage function found increased in LPS- compared with PIC-treated HBCs and vice versa. (C and D) Gene ontology (GO) analysis of the proteins up-regulated in (C) LPS-treated and (D) PIC-treated HBCs (FC > 1.5, adj. p value < 0.05). The proteins were analyzed by DAVID functional annotation to produce clusters (≥2 proteins/cluster) and GO terms corresponding to biological process (GOTERM_BP_FAT) were extracted. The histogram shows the top 20 GO terms significantly associated (adj p ≤ 0.05) with the protein list along with the number of proteins in each cluster. (E and F) Interaction networks of the up-regulated proteins in (E) LPS-treated and (F) PIC-treated HBCs using STRING v11.5. Colored lines between the proteins indicate the interaction evidence (pink: experimentally determined, blue: from curated databases), minimum required interaction score - medium confidence (0.4), disconnected nodes are hidden from the graph.

To explore the differences between bacterial and viral infections, we directly compared the protein abundances in the LPS and PIC-treated HBCs (Data S4). From the 360 differentially abundant proteins, 153 were increased in LPS- and 207 in PIC-treated HBCs (Figure 4A). The differentially abundant proteins consisted, amongst others, of chemokines, cytokines, chemokine/cytokine and scavenger receptors, HLA molecules, and other surface molecules (Figure 4B). LPS increased the levels of the pro-inflammatory chemokines CXCL1, CXCL3, CXCL5, CCL2, CCL3, and CCL20, while PIC induced CCL8, CXCL10, and CXCL11. HBCs treated with PIC were more abundant in IL18, an IFN-γ inducing factor, while LPS-treated HBCs were more abundant in IL6, IL8, IL1A, IL1B, IL36G, and TNF-α. Interestingly, the expression of HLA molecules including HLA-Α, HLA-B, HLA-C, HLA-F, and HLA-DRA was augmented in the PIC and reduced in the LPS group. The levels of several scavenger receptors were also different in the two treatment groups, with LPS-treated cells being more enriched in SCARF1, SCARB2, LRP1, MRC1, CD163, CD44, CD14, MSR1, CD209, CD36, COLEC12, CD163L1, CD68, and PIC-treated cells in MSR1 and CD68. Gene ontology analysis of the proteins increased in LPS, and decreased in PIC, indicated their involvement in biological processes including the regulation of the metabolic process, regulation of I-kappaB kinase/NF-κB signaling, production of molecular mediator of the immune response, cell adhesion and motility, and regulation of secretion (Figure 4C). Conversely, the proteins increased in PIC and decreased in LPS-treated HBCs, were involved in antigen processing and presentation of endogenous peptide antigen by MHC class I via ER pathway, TAP-independent viral process, regulation of cell adhesion, cell killing, cytokine production, defense response, and others (Figure 4D). Several interaction networks were observed in both LPS and PIC-treated HBCs (Figures 4E and 4F). The most represented Reactome pathways in LPS-treated HBCs were “Metallothioneins bind metals,” “Chemokine receptors bind chemokines,” and “Cholesterol biosynthesis.” In PIC-treated HBCs, pathway enrichment analysis revealed the induction of several Reactome pathways: “interferon alpha/beta signaling,” “interferon gamma signaling,” “ISG15 antiviral mechanism,” “antigen presentation: folding, assembly and peptide loading of class I MHC,” “endosomal/vacuolar pathway,” “DDX58/IFIH1-mediated induction of interferon-alpha/beta,” “negative regulators of DDX58/IFIH1 signaling,” “ER-phagosome pathway,” and “Negative regulators of DDX58/IFIH1 signaling” (Data S4). These data illustrate the distinct responses of HBCs to bacterial and viral ligands, even though some proteins might be commonly up or downregulated compared to the non-treated controls (Figure S2).

### Fetal sex differences in placental macrophage phenotype

**Figure 5:**
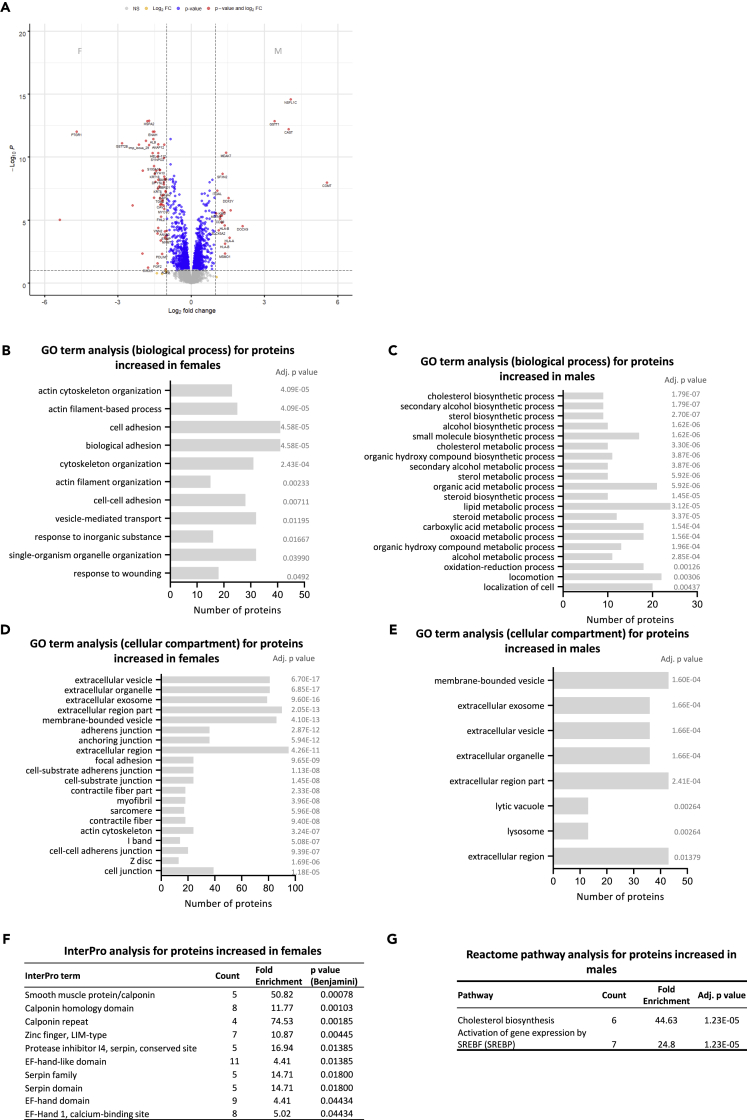
Sex-specific proteomic profiles of placental macrophages Differential expression analysis was performed in HBCs from female and male placentas, accounting for treatment, to identify differences due to sex. (A) Volcano plot depicting the fold differences in protein abundance between females (left) and males (right). (B and C) Gene ontology (GO) analysis, for the biological process, of the proteins increased (FC > 1.5, adj. p value < 0.05) in female HBCs compared with male HBCs (B) and of the proteins increased (FC > 1.5, adj. p value < 0.05) in male HBCs compared with female HBCs (C). (D and E) Gene ontology (GO) analysis, for the cellular compartment, of the proteins increased (FC > 1.5, adj. p value < 0.05) in female HBCs compared with male HBCs (D) and of the proteins increased (FC > 1.5, adj. p value < 0.05) in male HBCs compared with female HBCs (E). (F) Classification of protein families in the female HBC up-regulated proteins using Inter-Pro v86.0. (G) Reactome pathway enrichment analysis for the male HBC up-regulated proteins. For each pathway, the number of proteins (Count) and the (Fold Enrichment) are given.

We next sought to identify differences between HBCs of female and male fetuses by performing differential expression analysis, accounting for treatment, to identify differences due to sex (Figure 5A) (Data S5). The comparison identified 274 differentially abundant proteins. HBCs from female placentas were more abundant in proteins involved in actin and cytoskeleton organization, cell adhesion, and response to wounding (Figure 5B), while HBCs from male placentas were more abundant in proteins involved in lipid and alcohol metabolic processes and locomotion (Figure 5C). In both sexes, differentially abundant proteins were primarily extracellular region- and vesicle-associated (Figures 5D and 5E). Inter-Pro analysis showed that the female-specific proteins carried Calponin homology domains (TAGLN3, CNN3, TAGLN, FLNB, ACTN1, LMO7, TAGLN2, SPTBN1), Zinc finger motifs LIM-type (LMCD1, FHL2, LMO7, TGFB1, PDLIM7, PDLIM2, PDLIM1, PDLIM7), EF-hand(-like) domains (TPPP3, S100A1, RCN1, SPTAN1, S100P, S100A13, S100A16, ACTN1, CAPS, EHD2, PVALB), and a subset belonged to the Serpin family (SERPINB9, SERPINE2, SERPINA1, SERPINB2, SERPINH1) (Figure 5F). Reactome analysis of the male-specific proteins significantly identified just two pathways: “Cholesterol biosynthesis” (proteins involved: MSMO1, SQLE, HMGCS1, CYP51A1, FDFT1, HMGCR) and “Activation of gene expression by SREBF (SREBP)” (proteins involved: SQLE, DHCR7, HMGCS1, SC5D, CYP51A1, FDFT1, HMGCR) (Figure 5G). Sterol regulatory element-binding proteins (SREBP) are activators of transcription for genes involved in lipogenesis.^28^ These data suggest that sex-dependent differences in HBCs are primarily related to lipid metabolism in males and cytoskeleton organization in females.

### Fetal sex differences in placental macrophage responses to viral and bacterial pathogen-associated molecular patterns

**Figure 6:**
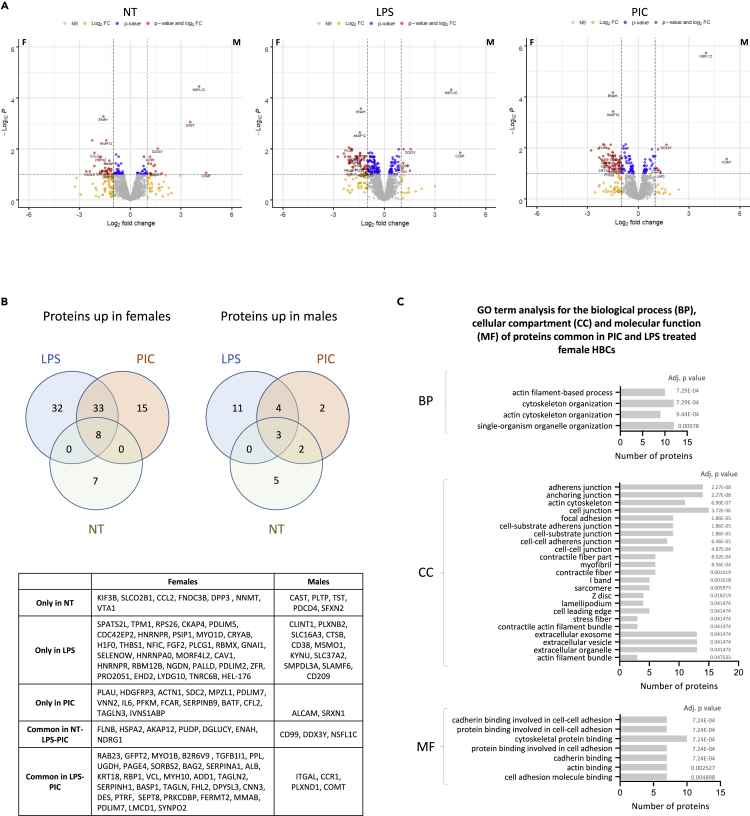
Sex-specific proteomic profiles of placental macrophages separated by treatment The proteomic profile of female and male HBCs was compared in each treatment group (NT, LPS, PIC). (A) Volcano plots depicting the fold differences in protein abundance between females (left) and males (right) in each treatment group separately NT, LPS, PIC (from left to right). (B) Venn diagrams of the differentially expressed proteins between female (left) and male (right) HBCs and their subclassification in the different treatment groups. The names of the proteins in each subgroup of the Venn diagrams are given in the table later in discussion. (C) Gene ontology (GO) analysis, for the biological process (BP), cellular compartment (CC), and molecular function (MF) of the 33 proteins common in PIC and LPS-treated female HBCs.

To explore sex-associated differences in HBC responses to bacterial and viral ligands, we next compared the proteome of female and male HBCs in each treatment group (NT, LPS, PIC) (Figure 6A) (Data S6). In the resting HBCs (NT), 15 proteins were significantly elevated in females compared with males and ten were higher in males (FC > 1.5 adj. p value < 0.05). In the LPS treatment group, 73 proteins were higher in females compared with males and 18 in males compared with females, while in the PIC treatment group, 56 proteins were higher in females and 11 in males (FC > 1.5 adj. p value < 0.05). We then proceeded to identify the male-female differentially abundant proteins that were common/disparate between the different treatment groups (Figure 6B). The proteins present in both PIC and LPS but not in the NT group revealed the sex-specific response to infection. Male HBCs expressed fewer proteins (ITGAL, CCR1, PLXND1, COMT) in response to LPS and PIC compared with female HBCs, which expressed proteins involved in actin filament and cytoskeleton organization, found primarily in cell junctions, and functioning as protein binding molecules (Figure 6C). In conclusion, HBCs exhibit intrinsic sex-associated differences that may affect their response to infection.

## Discussion

To investigate the response of placental macrophages to infection, HBCs were isolated from term placentas and exposed to bacterial LPS, a cell wall component of Gram-negative bacteria that signals primarily through toll-like receptor 4 (TLR4), or PIC a viral dsRNA analog that signals through TLR3. Quantitative proteomics was performed to comprehensively interrogate the phenotype of term HBCs, their response to bacterial and viral infection, and the contribution of fetal sex to these profiles.

We used tandem mass tag (TMT) proteomics, which has emerged as a novel high-throughput mass spectrometry method for protein identification and quantification in several experimental settings, including the description of monocyte surface markers.^29^ Here, a total of 5892 proteins were quantitatively identified in all three treatment groups (NT, LPS, and PIC). We showed that HBCs express a number of macrophage-related proteins, whose expression levels change in response to bacterial and viral PAMPs.

According to our data, term HBCs carry both classical and non-classical MHC class I, as well as MHC class II molecules, the expression of which is affected by bacterial and viral cues. A recent study by Thomas et al., suggested that first-trimester HBCs do not express HLA-DR (MHC class II) molecules and their presence may indicate contamination with placenta-associated maternal macrophages during isolation.^16^ However, ontogeny of early gestation and term HBCs is thought to be different, with early HBCs deriving from mesenchymal cells, while term HBCs may also include recruited yolk sac, fetal liver, and bone marrow monocytes.^2^^,^^30^ Importantly, in contrast to Thomas et al.,^16^ in which cells were isolated from just two placental enzymatic digests we used an optimised protocol which requires discarding the first three digests, which would contain contaminating cytotrophoblast and maternal blood cells, and only a fourth digest of the villous core was used for subsequent isolation followed by two negative immunoselection steps (using EGFR and then CD10 antibodies).^31^ We have also performed qPCR on our HBC isolations, for both female (XIST) and male (DDX3Y) markers, that confirms our expectation that we do not have significant amounts of contaminating maternal monocytes, as there is almost no XIST expression in the male HBCs. In fact, XIST levels in the highest XIST-expressing male were >300 times lower than those of the lowest XIST expressing female. Diversity in term HBCs might be due to their responses to the surrounding microenvironment such as senescent trophoblasts, necrotic tissue, and inflammatory cues.^32^ Though absent in early HBCs,^16^^,^^21^ HLA-DR may therefore be present in term HBCs, as reported previously.^33^^,^^34^

Upon LPS treatment, term HBCs switch toward a more pro-inflammatory phenotype, with increased M1 and decreased M2 phenotype markers. This is in agreement with previous studies reporting elevated secretion of the pro-inflammatory cytokines IL1B and/or TNF-α after LPS exposure of the first trimester^16^ and term HBCs.^14^ Swieboda et al., reported that term HBCs were less plastic than early or mid-gestational HBCs and predominantly M2-like,^35^ but we showed that term HBCs do respond to bacterial PAMPs by altering their expression profile. Our findings are supported by other studies documenting the capacity of term HBCs for pro-inflammatory responses.^36^^,^^37^ Some variability in the literature could be explained by the different methodological approaches utilized. For example, we analyzed HBC responses collectively using proteomics, while Schliefsteiner et al., used flow cytometry and showed that upon LPS and IFN-γ treatment, M2-like HBCs were reduced in number increasing the ratio of M1/M2 HBCs, suggesting that there is no shift in phenotype but rather a shift in the number of M2-like cells.^18^ Moreover, Young et al., using flow cytometry and immunohistochemistry showed that HBCs, though maintaining M2 marker expression, are exquisitely sensitive to LPS treatment.^36^ Similarly, Azari et al., showed that HBCs infected with the bacterial pathogen *Listeria monocytogenes* underwent pro-inflammatory reprogramming but maintained the expression of tolerogenic (M2 phenotype associated) genes.^38^

Upon PIC treatment, we observed some similar responses, where term HBCs expressed a plethora of pro-inflammatory cytokines and chemokines, including IL1, TNF-α, CCL3, CCL4, and CXCL10 among others. Other studies have shown that the treatment of HBCs with PIC increases the mRNA expression and secretion of the pro-inflammatory cytokines IL6 and IL8.^36^ HBCs have been demonstrated to both fight^17^^,^^39^^,^^40^ and permit^18^^,^^19^^,^^20^ the replication and transmission of viruses. The variability may lie with the viral species, the HBC donor, or the methodology.^41^ Our data clearly show the upregulation of antiviral pathways, such as IFN and ISG15 signaling, as well as overexpression of classical (HLA-A, HLA-B, HLA-C) and non-classical (HLA-F) MHC class I molecules in response to PIC.

After exploring the differences between treated and non-treated cells, we directly compared LPS and PIC-treated HBCs which highlighted the same pathways, showing that LPS and PIC have distinct effects on cells, as also seen by principal component analysis. HBCs, therefore, employ distinct defense mechanisms to fight bacterial and viral infections, as previously observed in human monocyte-derived macrophages, with LPS inducing NF-κB signaling and PIC elevating the expression of interferon-stimulated genes (ISG).^42^ However, we did identify some proteins that were similarly affected in LPS and PIC-treated HBCs, including common upregulation of both IL1 and TNF-α, as expected for these master regulators of inflammation.^11^

We next sought to investigate the role of fetal sex in the phenotype of placental macrophages. We identified >250 proteins differentially expressed between female and male HBCs. These included sex chromosome-associated proteins (DDX3Y upregulated in males and PUDP, PAGE4, PDLIM7, IL3RA, GK, PDLIM7, and EIF1AX in females), but also proteins encoded by genes on autosomes. Proteins more abundant in females were associated with actin and filament organization, whilst those more abundant in males were associated with metabolic processes including cholesterol (steroid) and alcohol biosynthesis. Others have also attempted to decipher sex differences between HBCs from first-trimester human^25^ and E17.5 (term) mouse (Caesarine et al., preprint)^43^ placentas using scRNAseq, however the number of differentially expressed genes identified in these studies was limited (17 and 27 respectively). Functional analysis of the differentially expressed genes between female and male murine HBCs showed distinct immune/defense responses and signaling via the JAK-STAT pathway (Caesarine et al., preprint).^43^ Regarding human HBCs, apart from the sex chromosome-associated genes, overexpression of the hemoglobin subunit β (HBB) in female HBCs was the only common finding between our study and Sun et al.,^25^ which might be due to the difference in gestation, or the type of material analyzed (RNA vs protein). The placenta-derived C-terminal fragment of HBB was previously reported to have antimicrobial properties, by blocking viral and/or bacterial entry, augmented in acidic (like inflammatory) conditions.^44^ We found that male HBCs had significantly higher levels of HLA-A and HLA-B compared with female HBCs, which might explain why male HBCs are more sensitive to pro-inflammatory cues *in utero*.^45^ For example, SARS-CoV-2 infection seems to affect disproportionally pregnancies with male fetuses, which exhibit increased placental inflammation and expansion of HBCs.^46^^,^^47^ Here, we observed that HBCs from females overexpressed several proteins that were associated with cytoskeleton and organelle organization, adhesion, and membrane configuration, in response to LPS and PIC. The motility of macrophages is important to facilitate their defense response, which requires reassembly of the actin meshwork for the polarization and formation of cell structures, like filopodia, and adhesion with the extracellular matrix.^48^ A subset of the proteins we found upregulated in female HBCs were calponins, which stabilise actin and are involved in signaling,^49^ and serpins, a superfamily of protease inhibitors with diverse roles including the inhibition of inflammatory response and hormone trafficking.^50^ We also found increased expression of intermediate filament proteins including vimentin, which can be located on the membrane, in the cytosol or extracellularly, in adjusting cells.^51^^,^^52^ In macrophages exposed to PAMPs or damage-associated molecular patterns (DAMPs) vimentin interacts directly with the intracellular sensor NLRP3 (NOD-, LRR- and pyrin domain-containing protein 3), triggering a pro-inflammatory response involving caspase-1.^53^ This mechanism was actually found to be adopted by HBCs in response to LPS and adenosine triphosphate (ATP),^14^ and our data now indicate that this might be sex specific.

We found that HBCs from female placentas mounted a much stronger response to bacterial and viral ligands compared to HBCs from male placentas, in terms of more increased proteins. There are many examples where adult females develop more potent immune responses than males, which result in increased vaccine efficiency and successful pathogen elimination, but also in their increased susceptibility to autoimmune diseases.^22^^,^^54^ In terms of innate immune responses, IFN-α production due to single-stranded DNA viruses is greater in females, based on exposure of blood samples to TRL7 ligands.^55^ Some immune-related differences between males and females are attributed to hormones, with transient increase in pro-inflammatory responses in males during puberty.^56^ Similarly, *in utero* immune dimorphism may be aided by the production of androgens from male testes from week 10 of gestation.^22^^,^^57^ Apart from hormones, environmental (nutrition and microbiota) and genetic mediators differentially influence immune responses in males and females *in utero* and throughout life (reviewed in Klein and Flanagan, 2016).^22^ It is reported that spontaneous abortion, pre-eclampsia and other pregnancy complications are more frequent in male fetuses.^57^^,^^58^^,^^59^

Overall, we have shown that HBCs respond to bacterial and viral ligands by altering their expression landscape to reflect a more pro-inflammatory phenotype, but separate mechanisms are employed based on the trigger. We also provide the first clear picture of sexual dimorphism in HBC proteomic profiles and responses to pathogens, primarily related to lipid metabolism in males and cytoskeleton organization in females, which could contribute to the observed relationship between fetal sex and responses to congenital and perinatal infections. To elucidate the sexual dimorphism at the maternal-fetal interface and its implications for the mother and the fetus during the various types of infection and inflammation further studies are needed. Moreover, as also suggested by Schliefsteiner et al.,^18^ and Thomas et al.,^16^ it may be time to acknowledge the pleiotropic nature of HBCs at the different stages of pregnancy and define specific placental macrophage phenotypes and signatures.

### Limitations of the study

We used a gold-standard HBC isolation method that grants high yield and purity, approximately 98% CD163+ cells, which show a highly vacuolated appearance, characteristic of HBCs *in vivo*,^31^^,^^36^ but we did not perform this purity analysis for every preparation in this current study. A possible limitation of this protocol could be contamination with blood monocytes that could differentiate into macrophages following plastic adherence, or with maternal macrophages adhered to the placental surface.^16^ In our protocol, only the fourth digest is used for HBC isolation, reducing the potential for contamination with maternal cells. We additionally tested for the presence of maternal cells, based on the detection of male and female genetic markers, as previously employed for sexing of placentas,^60^ finding no or very limited expression of the female marker XIST in male cell preparations, thereby demonstrating no significant contamination with maternal monocytes. Another method that could be employed in the future is a newer cell-sorting-based protocol that can remove contaminating maternal macrophages when there is an HLA mismatch between mother and fetus.^61^ One remaining limitation could be contamination with fetal-origin monocytes. However, the potential contamination is very small; the amount of blood on the final gradient indicates approximately 3 mL of contaminating blood from all sources (maternal intervillous space and fetal capillary) which could in theory contain approximately 400,000-900,000/mL monocytes.^62^ As we generally obtain about 200 million HBCs, there is therefore the potential for a <1% contamination with monocytes. This should be considered in this, and other, studies of HBCs or other tissue macrophages.

Another limitation of this study was that TMT proteomics currently allows only a maximum of 11 samples to be run in one batch. As our number of treatments and the inclusion of fetal sex necessitated the running of several batches, we were required to normalize between batches to enable robust analysis, with the caveat that we were only able to analyze proteins present in all three batches, resulting in some information loss. Another limitation, as a consequence of batch correction, is that only proteins that were present in all three treatment groups could be analyzed. However, the benefit of employing TMT-based quantitative proteomics meant that we were able to accurately quantify changes in protein levels between conditions, and perform paired analysis, which resulted in the identification of many more significant changes than could be obtained by non-quantitative proteomics. Additionally, while PAMPs are a widely used tool to simulate bacterial and viral infections in a controlled manner, they do not fully recapitulate real-life infection.^13^ Further functional experiments with whole viral and bacterial pathogens would strengthen the results of this study. Finally, the placenta donors in this study were exclusively White non-Hispanic individuals, which eliminates potential variability, but limits the findings of this study to a specific population. Our results should be validated in a larger cohort, including a range of ethnicities.

## STAR★Methods

### Key resources table

**Table:** 

REAGENT or RESOURCE	SOURCE	IDENTIFIER
**Antibodies**		

anti-EGFR	Santa Cruz	clone #528; RRID: AB_627492
anti-CD10	Biolegend	clone #HI10a; RRID: AB_314913

**Biological samples**		

Primary Hofbauer cells isolated from term placentas from women undergoing caesarean section	N/A	N/A

**Chemicals, peptides, and recombinant proteins**		

Trypsin	Gibco	Cat# 15090–046
DNase I	Roche	Cat# 10104159001
DNase I	Norgen	Cat# P4-0098 – 25710
Percoll	GE Healthcare Biosciences	Cat# 17-0891-01
RPMI-1640	Merk	Cat# R6504
Fetal bovine serum	GemCell™	Cat# 100–500
DMEM/F12	Merk	Cat#D2906
ITS + premix	Corning	Cat#354352
Lipopolysaccharides from *Escherichia coli* O 55:B5	Sigma	Cat# L2880
Poly(I:C) high molecular weight	Invivogen	Cat# tlrl-pic
RIPA buffer	Cayman Chemicals	Cat# 10010263
cOmplete Protease Inhibitor cocktail	Roche	Cat# 11873580001
PhosSTOP	Roche	Cat# 4906845001
Sso Advanced™ Universal Probes Supermix	Bio-Rad	Cat# 1725281
TMT reagents	Thermo Fisher Scientific	Cat# A34807

**Critical commercial assays**		

Micro BCA™ Protein Assay Kit	Thermo Fisher Scientific	Cat# 23235
Total RNA Purification Kit	Norgen	Cat# P4-0058-17200
High-Capacity RNA-to-cDNA™ Kit	Applied Biosystems	Cat# 4387406
Taqman expression assay for XIST (FAM-MGB)	Thermo Fisher Scientific	Hs01079824_m1
Taqman expression assay for DDX3Y (FAM-MGB)	Thermo Fisher Scientific	Hs00190539_m1
Taqman expression assay for GAPDH (VIC-MGB)	Thermo Fisher Scientific	Hs99999905_m1

**Deposited data**		

Proteomics raw data	PRIDE	PXD033006

### Resource availability

#### Lead contact

Further information and requests for resources and reagents should be directed to and will be fulfilled by the lead contact, Beth Holder (b.holder@imperial.ac.uk).

#### Materials availability

This study did not generate new unique reagents.

### Experimental model and subject details

#### Patient recruitment

Term placentas (≥37 weeks gestation) were obtained from uncomplicated singleton elective caesarean sections without labor performed at Yale New Haven Hospital. Human placental tissue collection was approved by Yale University’s Human Research Protection Program (IRB Protocol ID: 1208010742) and all samples were collected through the Yale University Reproductive Sciences (YURS) Biobank following patient consent. Exclusion criteria were: known multiple gestations and karyotypically abnormal fetuses, patients with chronic hypertension, pre-eclampsia, pregestational diabetes, systemic lupus erythematosus, autoimmune disease, congenital heart disease, chronic severe asthma, thrombophilic medical conditions, chronic abruption or vaginal bleeding during pregnancy and maternal infections (HIV, TB, malaria, SARS-CoV-2).

#### Hofbauer cell isolation and culture

Hofbauer cells (HBCs) were isolated using a standardised method (Figure S1A and S1B).^31^ Firstly, chorionic villous tissue from fresh placentas was minced, washed to remove blood, and sequentially digested with trypsin (Gibco, 15090-046) and DNase I (DNase I, Roche, 10104159001), to yield cytotrophoblasts: 15, 30 and 30 min. The trypsin-undigested tissue was then washed and digested with collagenase A (Roche, 11088793001) and DNAse I for 1h to obtain HBCs. Digests were passed through 100μm sieves and separated over a discontinuous Percoll (GE Healthcare Biosciences, 17-0891-01) gradient (35%/30%/25%/20%). Cells from the 20%/25–30%/35% interfaces were combined and stored in RPMI-1640 (Merk, R6504) supplemented with 5% fetal bovine serum (FBS, GemCell™, 100–500), 1% Pen-Strep, 4 mM L-glutamine, 25 mM HEPES overnight on ice. The following day, HBCs were further purified by negative immunoselection to deplete EGFR+ and CD10^+^ cells using antibodies conjugated to magnetic beads (anti-EGFR clone #528, Santa Cruz; anti-CD10 clone #HI10a, Biolegend). Fifteen million cells were plated in a 10cm^2^ culture dish in RPMI-1640 medium supplemented with 5% FBS, 1% Pen-Strep, 4 mM L-glutamine, 25 mM HEPES. After 1 h, any floating and weakly attached cells were removed with two washes of PBS and cells cultured in 10mL DMEM/F12 (Merk, D2906) supplemented with 10% FBS, 1% Pen-Strep, 4mM L-Glutamine. The following day, HBCs were washed twice in PBS and media exchanged for serum-free DMEM/F12 supplemented with 1% Pen-Strep, 4mM L-Glutamine, 50 μg/mL ascorbic acid and ITS + premix (Corning, 354352), yielding a final concentration of 6.25 μg/mL insulin, 6.25 μg/mL transferrin, 6.25 ng/mL selenous acid, 1.25 mg/mL BSA and 5.35 μg/mL linoleic acid.

Cells were left untreated or were treated with 1 ng/mL Lipopolysaccharides from *E. coli* O 55:B5 (LPS, Sigma) or with 1 μg/mL of the viral dsRNA mimic, Poly(I:C) (PIC, high molecular weight, Invivogen) for 24h. To demonstrate that our HBC preparations are free from maternal cells, we isolated RNA from HBCs and tested the expression of female (XIST) and male (DDX3Y) markers^60^ using real-time qPCR (Figure S1C).

### Method details

#### Protein preparation, quantitative tandem-mass tag (TMT) labeling and high pH reversed-phase chromatography

Cells were lysed with 750 μL 1x RIPA buffer (Cayman Chemicals) supplemented with protease and phosphatase inhibitors (cOmplete Protease Inhibitor cocktail and PhosSTOP, Roche). Lysates were incubated on ice for 15 min, centrifuged at 16,100 ×G for 10 min at 4°C, and the supernatant stored at −80°C until use. Protein was quantified using the Micro BCA™ Protein Assay Kit (Thermo Fisher Scientific). Samples were run in three batches for tandem mass tag (TMT) labeling: two 9-plex and one 11-plex, with the three treatments run on each batch (Figure S1). Aliquots of 50 μg of each sample were digested with trypsin (2.5 μg trypsin per 100 μg protein; 37°C, overnight), labeled with TMT ten/eleven plex reagents according to the manufacturer’s protocol (Thermo Fisher Scientific) and the labeled samples were pooled. An aliquot of 100 μg of the pooled sample was desalted using a SepPak cartridge according to the manufacturer’s instructions (Waters). Eluate from the SepPak cartridge was evaporated to dryness and resuspended in buffer A (20 mM ammonium hydroxide, pH 10) prior to fractionation by high pH reversed-phase chromatography using an Ultimate 3000 liquid chromatography system (Thermo Fisher Scientific. In brief, the sample was loaded onto an XBridge BEH C18 Column (130Å, 3.5 μm, 2.1 mm × 150 mm, Waters, UK) in buffer A and peptides eluted with an increasing gradient of buffer B (20 mM Ammonium Hydroxide in acetonitrile, pH 10) from 0–95% over 60 min. The resulting fractions were evaporated to dryness and resuspended in 1% formic acid prior to analysis by nano-LC MSMS using an Orbitrap Fusion Lumos mass spectrometer (Thermo Scientific).

#### Nano-LC mass spectrometry

High pH RP fractions were further fractionated using an Ultimate 3000 nano-LC system in line with an Orbitrap Fusion Lumos mass spectrometer (Thermo Scientific). In brief, peptides in 1% (v/v) formic acid were injected onto an Acclaim PepMap C18 nano-trap column (Thermo Scientific). After washing with 0.5% (v/v) acetonitrile 0.1% (v/v) formic acid peptides were resolved on a 250 mm × 75 μm Acclaim PepMap C18 reverse phase analytical column (Thermo Scientific) over a 150 min organic gradient, using 7 gradient segments (1–6% solvent B over 1min, 6–15% B over 58 min, 15–32% B over 58 min, 32–40% B over 5 min, 40–90% B over 1 min, held at 90% B for 6 min and then reduced to 1% B over 1 min) with a flow rate of 300 nL/min. Solvent A was 0.1% formic acid and Solvent B was aqueous 80% acetonitrile in 0.1% formic acid. Peptides were ionized by nano-electrospray ionization at 2.0 kV using a stainless-steel emitter with an internal diameter of 30 μm (Thermo Scientific) and a capillary temperature of 300°C.

All spectra were acquired using an Orbitrap Fusion Lumos mass spectrometer controlled by Xcalibur 3.0 software (Thermo Scientific) and operated in data-dependent acquisition mode using an SPS-MS3 workflow. FTMS1 spectra were collected at a resolution of 120,000, with an automatic gain control (AGC) target of 200,000 and a max injection time of 50 ms. Precursors were filtered with an intensity threshold of 5000, according to charge state (to include charge states 2–7) and with monoisotopic peak determination set to Peptide. Previously interrogated precursors were excluded using a dynamic window (60s +/−10ppm). The MS2 precursors were isolated with a quadrupole isolation window of 0.7 *m*/*z*. ITMS2 spectra were collected with an AGC target of 10,000, max injection time of 70 ms and CID collision energy of 35%.

For FTMS3 analysis, the Orbitrap was operated at 50,000 resolution with an AGC target of 50,000 and a max injection time of 105 ms. Precursors were fragmented by high energy collision dissociation (HCD) at a normalised collision energy of 60% to ensure maximal TMT reporter ion yield. Synchronous Precursor Selection (SPS) was enabled to include up to 10 MS2 fragment ions in the FTMS3 scan.

#### Protein identification and quantification

The raw data files were processed and quantified using Proteome Discoverer software v2.1 (Thermo Scientific) and searched against the UniProt Human database (downloaded August 2020: 167789 entries) using the SEQUEST HT algorithm. Peptide precursor mass tolerance was set at 10ppm, and MS/MS tolerance was set at 0.6Da. Search criteria included oxidation of methionine (+15.995 Da), acetylation of the protein N-terminus (+42.011Da) and Methionine loss plus acetylation of the protein N-terminus (−89.03 Da) as variable modifications and carbamidomethylation of cysteine (+57.021 Da) and the addition of the TMT mass tag (+229.163 Da) to peptide N-termini and lysine as fixed modifications. Searches were performed with full tryptic digestion and a maximum of 2 missed cleavages were allowed. The reverse database search option was enabled and all data was filtered to satisfy false discovery rate (FDR) of 5%.

#### RNA extraction

Total RNA was isolated from RIPA lysates using a Total RNA Purification Kit (Norgen, P4-0058 – 17200). Briefly, 50μL of the lysate were mixed with 300μL of Buffer RL followed by the addition of 200μL absolute ethanol. The mixture was then passed through Mini Spin Columns, washed, DNAse I treated (Norgen, P4-0098 – 25710), and eluted in 30μL elution solution. RNA concentration and integrity were assessed on an Agilent 2100 Bioanalyzer system using an RNA 6000 nano chip (Agilent).

#### Reverse transcription

200 ng of RNA were used to create a cDNA library using the High-Capacity RNA-to-cDNA™ Kit (Applied Biosystems, 4387406) following the manufacturer’s instructions. After completion of cDNA synthesis, 30 μL of nuclease free water was added in each sample.

#### Real-time qPCR

Real-time qPCR was performed using the Sso Advanced™ Universal Probes Supermix (Bio-Rad, 1725281) and the Taqman Gene Expression Assays GAPDH (Hs99999905_m1, VIC-MGB), XIST (Hs01079824_m1, FAM-MGB), and DDX3Y (Hs00190539_m1, FAM-MGB). The reactions were duplexed (GAPDH and gene of interest) and were run on an STEP-ONE real-time PCR system (Applied biosystems, 4376357). We mixed equal quantities of female and male placenta cDNA, which we run along with our samples, to serve as a calibrator, as it expressed both XIST and DDX3Y. The data were analyzed using the ΔΔCt method and the relative expression was calculated as 2^−ΔΔCt^.

### Quantification and statistical analysis

For the proteomics analysis, the normalised abundance values were imported into R for statistical analysis.^63^ The abundance values were log_2_ transformed and only proteins that were measured across all three batches were used for subsequent analysis. Batch effect adjustment was undertaken using SVA^64^ and Principal Component Analysis using PCATools^65^ (Figure S1D). Subsequent differential expression analysis was conducted using limma, fitting linear models in a paired manner, while accounting for treatment, sex and batch.^66^ p values were adjusted for multiple testing. Enhanced volcano was used for volcano plot visualisations.^65^

Gene ontology (GO) enrichment analysis of the generated datasets was performed using DAVID Bioinformatics Resources 6.8 with the default background.^67^^,^^68^ Protein functions were classified into three subgroups: biological process (BP), cellular compartment (CC), and molecular function (MF). The subset of the more specific terms represented by the GO_Fat categories was selected for further analysis. The genomic sources Reactome 78.0^69^ and Inter-Pro 87.0^70^ were used to identify enriched pathways and protein families in each dataset, respectively. The Benjamini correction was used to assess statistical significance. Enriched GO terms, Reactome pathways, and Inter-Pro entries with adj. p value < 0.05 were considered statistically significant. Interaction networks were produced in STRING v11.5 using experimentally determined interactions and interactions arising from curated databases, and the minimum required interaction score - medium confidence (0.4).^71^

## Data Availability

• The mass spectrometry proteomics data PRIDE:PXD033006 are publicly available as of the date of publication. Metadata and lists of proteins are available as supplementary data. • No original code was generated in this study. • Any additional information required to reanalyze the data reported in this paper is available from the lead contact upon request.

## Supplemental information

Document S1. Figures S1, S2 and Table S1

Data S1. All HBCs, related to Figure 1

Data S2. LPS-NT, related to Figure 2

Data S3. PIC-NT, related to Figure 3

Data S4. LPS-PIC, related to Figure 4

Data S5. Sex differences, related to Figure 5

Data S6. Sex response, related to Figure 6
