# Supplementary material for: Placental macrophage responses to viral and bacterial ligands and the influence of fetal sex
Source: iScience. 2022 Nov 22;25(12):105653. doi: 10.1016/j.isci.2022.105653 (PMC9732417; doi:10.1016/j.isci.2022.105653)
Supplement: Document S1. Figures S1, S2 and Table S1 [file mmc1.pdf]

iScience, Volume *25*

Supplemental information

Placental macrophage responses to viral and bacterial ligands and the influence of fetal sex

Paschalia Pantazi, Myrsini Kaforou, Zhonghua Tang, Vikki M. Abrahams, Andrew McArdle, Seth Guller, and Beth Holder

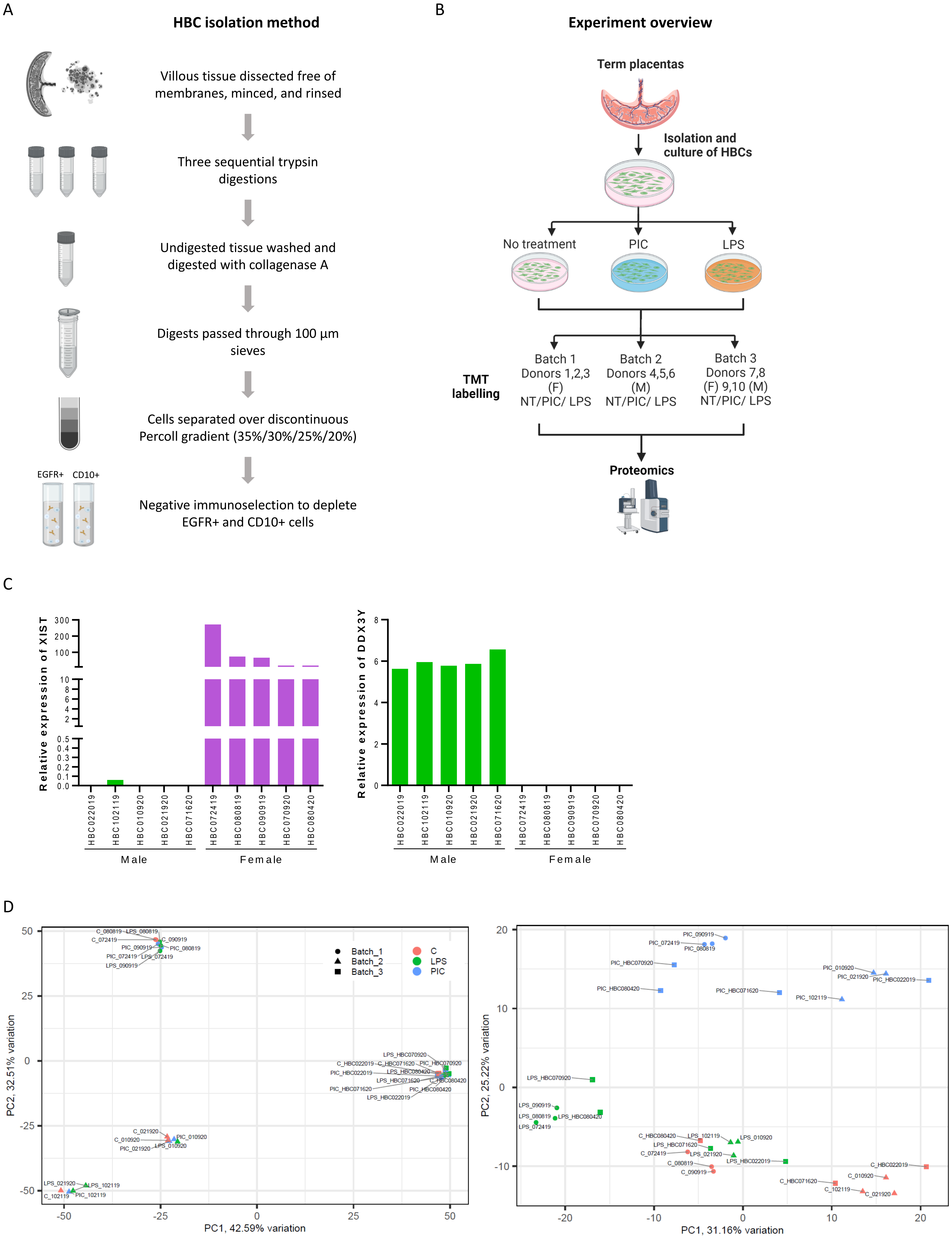
A
HBC isolation method
Villous tissue dissected free of membranes, minced, and rinsed
Three sequential trypsin digestions
Undigested tissue washed and digested with collagenase A
Digests passed through 100 µm sieves
Cells separated over discontinuous Percoll gradient (35%/30%/25%/20%)
EGFR+
CD10+
Negative immunoselection to deplete EGFR+ and CD10+ cells
B
Experiment overview
Term placentas
Isolation and culture of HBCs
No treatment
PIC
LPS
TMT labelling
Batch 1 Donors 1,2,3 (F) NT/PIC/ LPS
Batch 2 Donors 4,5,6 (M) NT/PIC/ LPS
Batch 3 Donors 7,8 (F) 9,10 (M) NT/PIC/ LPS
Proteomics
C
Relative expression of XIST
Relative expression of DDX3Y
HBC022019
HBC102119
HBC010920
HBC021920
HBC071620
HBC072419
HBC080819
HBC090919
HBC070920
HBC080420
Male
Female
D
Batch_1
Batch_2
Batch_3
C
LPS
PIC
PC1, 42.59% variation
PC2, 32.51% variation
PC1, 31.16% variation
PC2, 25.22% variation

Figure S1. Related to STAR methods

Experimental design and data normalisation. A) Hofbauer cells (HBCs) were isolated from term placentas using enzymatic digestion, percoll gradients and negative immunoselection. B) HBC were cultured for 24h in the presence and absence of viral or bacterial ligands. Lipopolysaccharide (LPS) and poly(I:C) (PIC) were used to simulate bacterial and viral infection, respectively. Cell protein was extracted and analysed using a tandem mass tag (TMT) proteomics approach, whereby all samples are labeled with isobaric tags and mixed together. Labeled peptides were fractionated, subjected to LC-MS/MS, and data analyzed using R. C) To ensure that the HBC preparations were free from maternal cells, RNA was isolated from HBC protein lysates, to investigate the expression of female (XIST – left) and male (DDX3Y – right) genetic markers using real-time qPCR. Samples from female placentas are indicated with the initial “F” and males with “M". We detect almost no expression of XIST in the male samples, indicating that there is no significant contamination with maternal cells. The expression of DDX3Y solely by the male HBCs confirms the sex phenotype. D) Principal Component Analysis plots before (left) and after (right) batch correction. The first and the second principal components are plotted here.

A

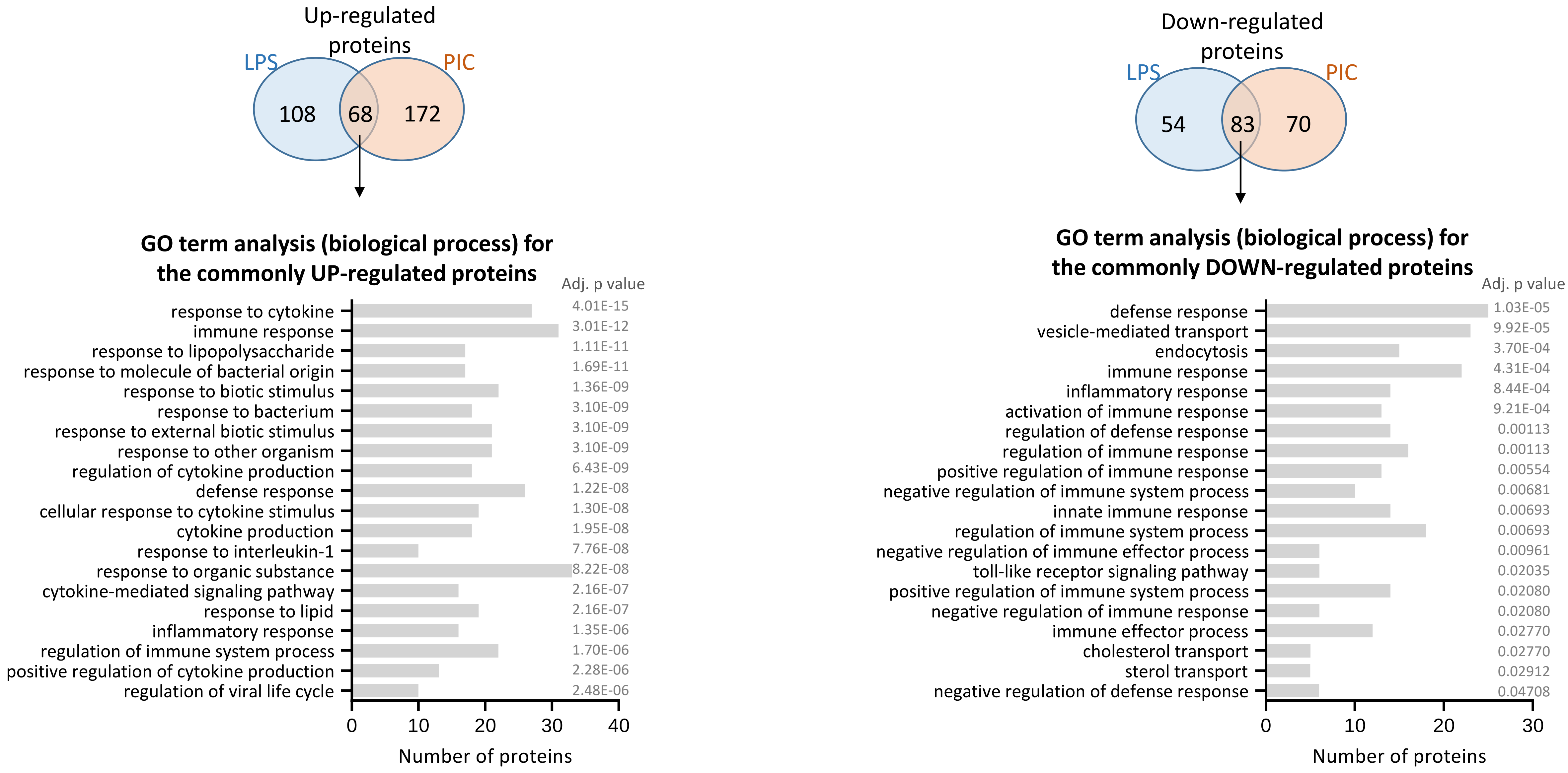

B

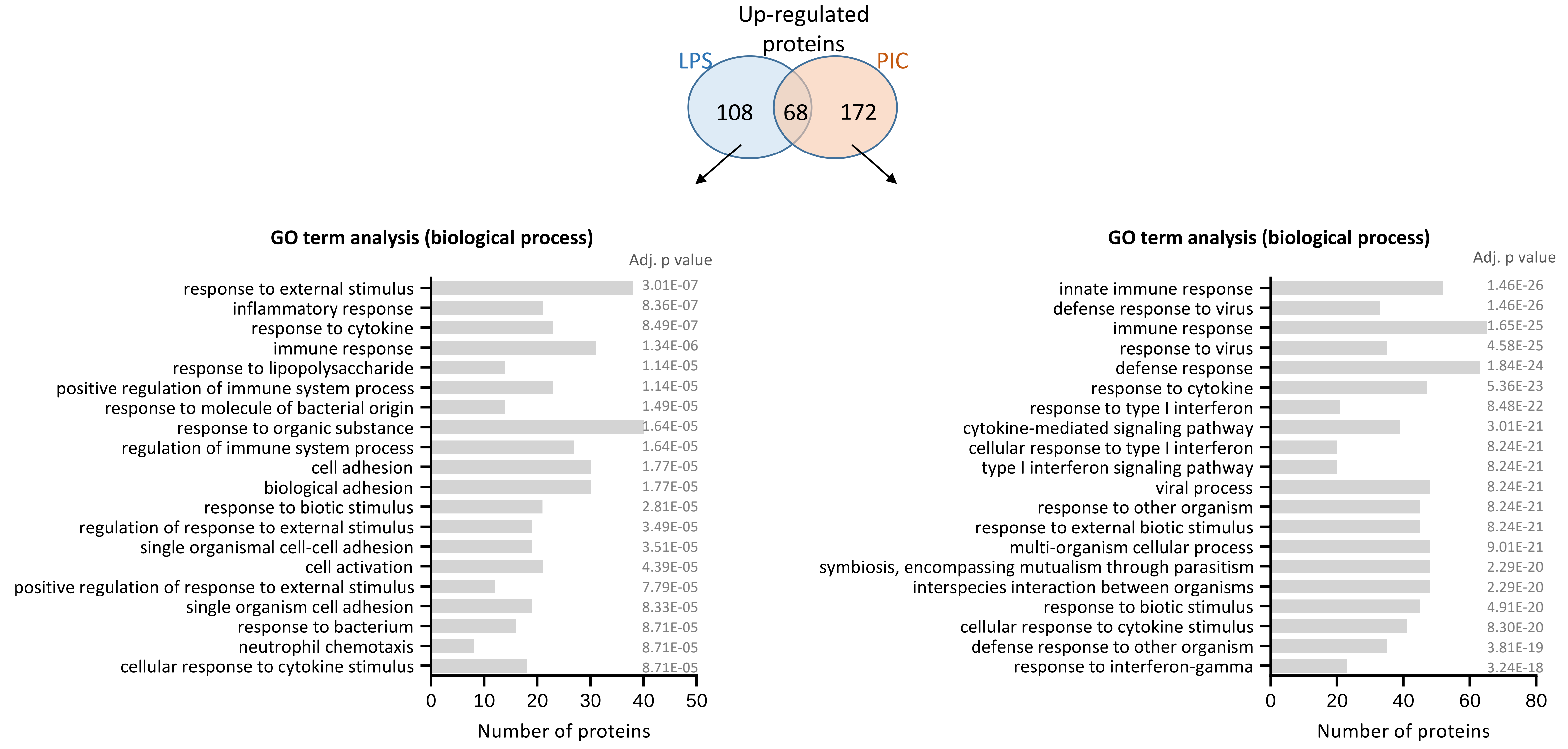

Pathway	Count	Fold Enrichment	p value (Benjamini)
Metallothioneins bind metals	4	47.83	0.00492
Chemokine receptors bind chemokines	6	13.38	0.00492

Pathway	Count	Fold Enrichment	p value (Benjamini)
Interferon alpha/beta signaling	20	29.13	5.87E-21
Interferon gamma signaling	17	18.85	2.49E-14
ISG15 antiviral mechanism	10	13.37	2.89E-06
Antigen Presentation: Folding, assembly and peptide loading of class I MHC	7	26.27	9.00E-06
Endosomal/Vacuolar pathway	5	40.66	1.84E-04
ER-Phagosome pathway	8	9.29	7.00E-04
DDX58/IFIH1-mediated induction of interferon-alpha/beta	4	22.96	0.01767

Figure S2. Related to Figures 2 and 3.

Common and differential placental macrophage responses to bacterial and viral PAMPS. The proteins that were up-regulated in LPS vs NT (FC>1.5, adj p value <0.05) were compared with the proteins that were up-regulated in PIC vs NT (FC>1.5, adj p value <0.05). A) Venn diagram showing the overlap between the up- and down-regulated proteins in LPS and PIC-treated HBCs, and Gene ontology (GO) analysis of the commonly up-regulated proteins in LPS and PIC –treated HBCs. B) Venn diagram showing the proteins that were up-regulated only in the LPS and only in the PIC treated HBCs, and GO analysis along with Reactome pathway analysis of these proteins.

Supplemental Table 1. Related to STAR methods. Patient information for the ten included placentas (w+d; weeks and days).

Gestational age (w+d)	Ethnicity	Fetal sex
**39+1**	White, non-Hispanic	Male
**39+1**	White, non-Hispanic	Male
**39+2**	White, non-Hispanic	Male
**39+4**	White, non-Hispanic	Male
**39+1**	White, non-Hispanic	Male
**UNK**	White, non-Hispanic	Female
**UNK**	White, non-Hispanic	Female
**UNK**	White, non-Hispanic	Female
**39+0**	White, non-Hispanic	Female
**39+0**	White, non-Hispanic	Female
